# Rejuvenation of Mesenchymal Stem Cells to Ameliorate Skeletal Aging

**DOI:** 10.3390/cells12070998

**Published:** 2023-03-24

**Authors:** Mingjia Cheng, Weihao Yuan, Alireza Moshaverinia, Bo Yu

**Affiliations:** 1 Section of Restorative Dentistry, School of Dentistry, University of California, Los Angeles, CA 90095, USA; 2 Section of Advanced Prosthodontics, School of Dentistry, University of California, Los Angeles, CA 90095, USA

**Keywords:** stem cells, aging, bone regeneration, senescence

## Abstract

Advanced age is a shared risk factor for many chronic and debilitating skeletal diseases including osteoporosis and periodontitis. Mesenchymal stem cells develop various aging phenotypes including the onset of senescence, intrinsic loss of regenerative potential and exacerbation of inflammatory microenvironment via secretory factors. This review elaborates on the emerging concepts on the molecular and epigenetic mechanisms of MSC senescence, such as the accumulation of oxidative stress, DNA damage and mitochondrial dysfunction. Senescent MSCs aggravate local inflammation, disrupt bone remodeling and bone-fat balance, thereby contributing to the progression of age-related bone diseases. Various rejuvenation strategies to target senescent MSCs could present a promising paradigm to restore skeletal aging.

## 1. Introduction

Skeletal aging is a multifactorial deterioration of bony architecture associated with reduced bone density and increased fracture risk. Advanced age is a shared risk factor for many chronic and debilitating skeletal diseases including osteoporosis and periodontitis [1,2]. Osteoporosis is a multifactorial, age-related progressive bone disorder that afflicts over 200 million worldwide [3]. Emerging evidence suggests that intrinsic aging-related mechanisms are at play in the pathogenesis of osteoporosis, including dysregulated osteoimmune responses and aberrant stem cell lineage allocation [4,5,6,7]. Beyond the systemic bone disease, the aging of the craniofacial skeleton, which is more pronounced in the mandible and alveolar processes [8], significantly impedes the repair of trauma-induced bony defects [9], and complicates treatment outcomes affecting dental implants and facial esthetics [10]. Periodontitis is the inflammatory destruction of the alveolar bone and periodontal connective tissue, resulting in the loss of tooth support [11]. The susceptibility and severity of periodontitis increase dramatically with age, as prevalence of severe periodontitis doubles in adults 65 or older [12]. As the age-related reduction of systemic bone mineral density (BMD) in the axial and appendicular skeleton is also associated with lower BMD in the alveolar bone [13,14,15], skeletal aging could contribute to the age-induced exacerbation of periodontitis. While aging promotes pathogenic microbial colonization and evokes a pro-inflammatory microenvironment to exacerbate periodontal inflammation and bone loss [1,2,16], new evidence points to the development of aging phenotypes in the mesenchymal stem cells that could also play an important role in the progression and exacerbation of periodontitis in elderly patients. In both osteoporosis and periodontitis, these new pathogenic mechanisms converge in the age-altered interactions between the marrow progenitor cells with their surrounding microenvironment.

Mesenchymal stromal/stem cells (MSCs) are multipotent stromal and progenitor cells with a self-renewal property and a differentiation ability in multiple cell types, such as bone, cartilage, adipose, and tendon cells [17,18]. Characterization of MSCs from tissue-specific sources and their interactions with surrounding cell types in the tissue microenvironment are essential to harness their potential in regenerative medicine. Under extrinsic and intrinsic factors, adult MSCs undergo aging and display a number of well-characterized aging phenotypes associated with a marked decline in regenerative potential [19]. The interactions of aging and senescent MSCs within the tissue environment may contribute to the exacerbation of the immune response that aggravates inflammation-driven bone disorders such as osteoporosis and periodontitis and could form novel therapeutic targets for these prevalent diseases [20,21]. This review elaborates on the emerging concepts linked to MSC senescence and the development of age-related bone diseases, subsequently shedding light on potential therapeutic approaches to rejuvenate aged MSC to restore skeletal aging.

## 2. Aging Phenotypes of MSCs

Cellular senescence, the halting of proliferation for damaged and dysfunctional cells, is critical in the pathogenesis of age-related chronic diseases including diabetes and osteoporosis [22,23,24,25]. MSCs, particularly those derived from the bone marrow (BMSCs), develop senescence as a hallmark of their aging process, whether through physiological stress in vivo or through replicative passaging in vitro [26]. Replicative senescence appears to be an irreversible arrest of cell proliferation due to the Hayflick limit [27]. Premature senescence happens at an earlier stage when cells undergo stress, such as oxidative stress, suboptimal culture conditions, and exposure to DNA damage [26]. The negative impact of MSC senescence on tissue homeostasis is twofold: the intrinsic exhaustion of the MSC pool, and the altered modulation of the immune microenvironment. The main characteristics of senescent BM-MSCs include halted proliferation, reduced self-renewal and impaired differentiation, leading to “stem cell exhaustion” in vivo [28] and contributing to the impaired bone mass and delayed repair in long-bone [29,30]. Cellular senescence is also associated with inflammation and extracellular matrix remodeling through the secretion of proteins termed as senescence-associated secretory phenotype (SASP) [31]. The pro-inflammatory microenvironment would in turn inhibit osteoblasts and promote osteoclasts, resulting in the disruption of balanced bone remodeling and a net bone loss [32]. The loss of homeostasis maintenance further impairs tissue regeneration capacity, exacerbating age-related degeneration and diseases in response to stress and injury [33]. We will summarize below the key aging phenotypes associated with MSC senescence.

### 2.1. Changes in Cell Morphology

Primary MSCs in the early passage culture (Passage 1 to 3) display small, uniform and spindle shapes, but develop up to a five-fold enlarged and flat morphology after Passage 5 [34,35]. A similar increase in cell size is also correlated to donor age in primary MSCs [36]. Larger cell size has been associated with differentiation potential [37], auto-fluorescence and metabolism. While increased cell size is linked to a decrease in multipotency [38] and mineralization potential [39], the enhanced auto-fluorescence in larger cells has been proposed as a potential biomarker for cellular senescence [40]. It has been suggested that the lack of cell size uniformity could be auto-corrected via an adjustment in cell cycle length and growth rate [41], therefore the loss of uniformity in the late-passage culture could be indicative of an abnormal cell cycle progression.

### 2.2. Proliferation and Self-Renewal Ability

The enlargement in aged MSC cell size is accompanied with a decline in proliferative capacity. MSCs from young donors possess close to a 1.5-fold increase in mitotic activity, and almost double the proliferation rate compared to those from old donors [42,43]. Cellular senescence is characterized by the growth arrest in the G1 phase of the cell cycle. In senescent BMSCs, growth arrest leads to a reduced self-renewal frequency. BMSCs in vivo are usually in a semi-quiescent state, which means replicative exhaustion is a secondary cause during natural aging in vivo [44]. Senescence can lead to a decrease in the stem cell pool by reducing the self-renewal ability of BMSCs [26]. The most common way of testing the self-renewal ability of MSCs is the colony-forming unit fibroblast (CFU-F) assay. DiGirolamo et al. [45] were the first to find that the colony-forming efficiency of normal BMSCs decreased dramatically using CFU-f assay when they were expanded from passage 2 to passage 7. The reduced fibroblastic colony-forming unit number and the average colony size are correlated with MSC aging and senescence in vitro [46].

### 2.3. Reduced MSC Migration and Homing

The directed migration and homing of MSCs to injury sites is critical for tissue regeneration. MSCs derived from old donors possess a less dynamic reorganization of the actin cytoskeleton in response to biological and mechanical cues [47], which in turn reduces cell migration [48,49]. Furthermore, the gene expression of several chemokines and their receptors essential to regulate cell migration are also significantly reduced in aged MSCs, including stromal cell-derived factor 1 (SDF-1), chemokine receptor type 4 (CXCR4), tumor necrosis factor receptor (TNFR) and C-C motif chemokine receptor 7 (CCR7) [50,51]. Thus, senescent MSCs display a reduced migration ability [49].

### 2.4. Changes in Differentiation Potentials

MSC senescence is responsible for a decrease in the overall multipotent differentiation abilities. The osteogenic potential of BMSCs decreases with senescent cell accumulation [52]. There is also a shift in lineage commitment for aged MSCs. The predominant belief is that aged MSCs strongly favor adipogenic over osteoblastic differentiation both in vitro and in vivo [53,54,55] although, several reports contrast to this trend where both lineage potentials are reduced with age [51]. This discrepancy could be due to the tissue source and the in vitro induction conditions. Oxidative stress and epigenetic stress are possible fate decision inducers [5,56]. MSC senescence plays a critical role in the switch between the osteogenetic and adipogenetic commitment by controlling a number of transcription factors and signaling pathways that mediate osteogenesis and adipogenesis. While osteogenic master regulators such as runt-related transcription factor2 (RUNX2) are downregulated with age or passage, adipogenic master regulators such as peroxisome proliferator-activated receptor γ (PPARγ) are upregulated [57]. In addition, several signaling pathways regulating the MSC cell fate have also been implicated as being influenced by aging, including Wnt/β-catenin, Indian Hedgehog (IHH) and TGF-β pathways [58,59,60,61]. Besides altered signaling pathways, extracellular vesicles also play a role in this transition. Recently, Wang et al. reported that the extracellular vesicles derived from the aged bone matrix during bone resorption promoted the adipogenesis of BM-MSCs rather than osteogenesis and increased vascular calcification [62]. Taken together, the aberrant lineage allocation of MSCs, via assay of the mutually exclusive cell fate, is also used as an indirect indicator for MSC senescence.

### 2.5. SASP and Inflammation

The word “inflamm-aging” refers to the relationship between inflammation and the aging process because they promote each other. Increased levels of pro-inflammatory cytokines are observed in elderly and osteoporotic patients [63]. Well-controlled inflammatory levels during bone injury are supportive for bone healing [64]. However, a persistent proinflammatory environment is reported to promote senescence and favor adipogenic differentiation of BMSCs to causes bone loss [65]. The exogenous TGF-β expression could trigger premature senescence in BMSCs [60], while inhibiting the TGF-β receptor signaling was found to be beneficial for the expansion of undifferentiated MSCs [66].

Senescent cells secrete a range of proinflammatory cytokines, chemokines, proteases, and growth factors. These are termed the senescence-associated secretory phenotype (SASP). This acquired aging phenotype reflects the dysregulated autocrine/paracrine interactions between MSCs with the surrounding cells, to exacerbate the proinflammatory microenvironment in aged tissue. Aged MSCs reportedly possess a diminished ability to suppress allogenic peripheral blood mononuclear cells compared to young MSCs [67], while proinflammatory cytokines such as interleukins (IL-1α, IL-6) are significantly upregulated in the conditioned medium from aged MSCs [68,69]. These SASP factors are crucial to reinforce the final senescent process of MSCs, playing key roles in stress-responsive, cytoprotective and immunoregulatory activities in the bone marrow. Finally, the inflammatory microenvironment caused by SASP from senescent MSCs can accelerate the senescence or induce apoptosis of neighboring cells [70].

### 2.6. ECM Stiffness and Reduced Extracellular Matrix Turnover

Aging-related extracellular matrix (ECM) alterations such as impaired collagen remodeling and mineralization balance are further consequences of MSC senescence, leading to ECM stiffness. An aged ECM microenvironment in turn causes the impaired proliferative and osteogenic potential of BMSCs, thus creating a vicious cycle. Both mouse and human in vitro studies have demonstrated that aged BMSCs cultured on young ECM have enhanced proliferative and osteogenic capacities compared with old ECM [71,72].

## 3. Molecular Mechanisms of MSC Senescence

**Figure 1:**
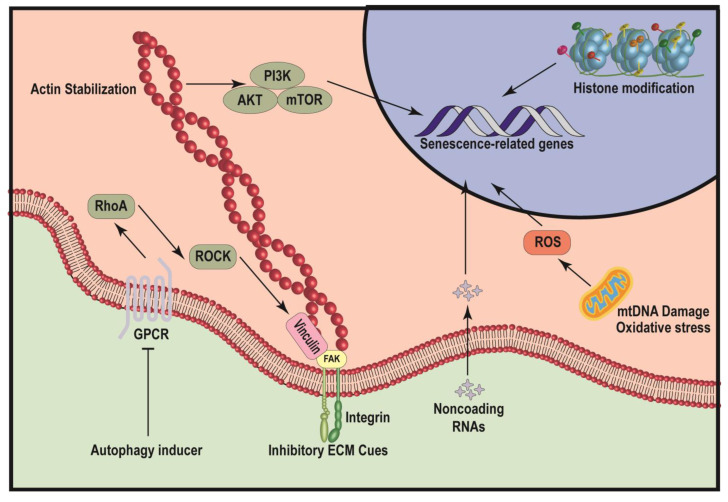
Schematic illustration of mechanisms leading to MSC senescence. The diagram illustrates several pathways that can induce cellular senescence, including autophagy, inhibitory ECM cues, noncoding RNAs, histone modification and mitochondria dysfunction. Both autophagy inducer and inhibitory ECM cues repress the function of focal adhesion complex, thereby inhibiting the mechanotransduction-mediated actin stabilization and downstream PI3K/AKT/mTOR signaling pathway and finally induce the expression of senescence-related genes. Noncoding RNAs and histone modifications can modulate the accessibility of senescence-related genes to alter genetic programing regulating senescence. mtDNA damage and oxidative stress of mitochondria can significantly promote the production of ROS, to induce the expression of senescence-related genes.

MSC senescence is a complex and progressive process involving several key age-related mechanisms. Several well-studied mechanisms are summarized below (Figure 1).

### 3.1. ROS Accumulation and Oxidation Prevention

Oxidative stress is one of the main causes of chronic senescence. An age-related increase of oxidative stress can be caused by a reduced endogenous antioxidants production, chronic inflammation, mitochondria dysfunction and mitophagy defection. Based on the free radical theory of aging, the most well-studied age-related accumulation of toxic metabolite in bone marrow-derived MSCs is the reactive oxygen species (ROS). The production of the ROS at a normal level plays an essential role in cell signaling and homeostasis. However, ROS levels can increase dramatically under environmental stress, resulting both from an uncontrolled production and from an inefficient elimination, and may severely damage the BMSCs. A number of factors can lead to an elevated ROS level in BMSCs. Recently, NADH dehydrogenase (ubiquinone) iron-sulfur protein 6 (Ndufs6) deficiency with age is reported to increase intracellular and mitochondrial ROS in BMSCs [73]. Other regulators include IFN-γ, tuberous sclerosis complex 1 (TSC1), type 1 interferon (IFN1) signaling and Indian hedgehog (IHH) [65,74,75]. Together, these studies suggest that intracellular accumulation of the ROS contributes to the aging of BMSCs and can be reversed by antioxidant methods, which in turn can influence BMSC senescence and stemness.

### 3.2. LPO and Lipid Metabolism

Besides direct damage caused by the ROS, when oxidants attack lipids that contain C–C double bonds, particularly polyunsaturated fatty acids (PUFAs), lipid peroxidation (LPO) would be induced. The accumulation of oxidative degradation of lipids results in cell damage and the aging process. Since adipose tissue increases in long bone marrow with age, LPO can also promote BMSCs’ aging. Moreover, aging BMSCs have altered membrane lipid composition and functionality [76]. An analysis of age-related metabolomics and transcriptomics reveals differential metabolites abundant in glycerophospholipid metabolism, linoleic acid metabolism and the biosynthesis of unsaturated fatty acids [77]. Results also indicated that the differential genes related to lipid metabolism may be closely associated with the aging of BMSCs. Another age-related lipidomics and transcriptomics analysis on changes of lipids and pathways demonstrated that the changing trends and significances of lipids during passaging were associated with the chain length and the degree of unsaturation in human BMSC [78]. D6 (distillate 6), by-products of the olive oil generation, is rich in squalene. A diet supplement with D6 reduced lipidic peroxidation in the serum of postmenopausal women. An in vitro study showed that human BMSC cultured with such serum had increased osteoblastogenesis and reduced adipogenesis [79].

### 3.3. Protein Homeostasis Disruption and Proteostasis Regulators

Protein homeostasis (proteostasis) is known as the network that maintains proteins in the correct state during protein synthesis, folding and turnover. Impaired protein homeostasis with age results in endoplasmic reticulum (ER) stress, leading to inflammation, cellular senescence and apoptosis. For example, prelamin A is an abnormally processed form of the nuclear lamina protein lamin A. Prelamin A accumulation can trigger the premature senescence of subchondral bone mesenchymal stem cells (SCB-MSCs) by inducing DNA damage, which can be rescued by vitamin C (VC) treatment [80]. Age is one of the main risk factors that can cause proteostasis defects and accumulation of misfolded and damaged proteins, leading to degenerative diseases such as Alzheimer’s disease. A number of studies have explored the role of autophagy in BMSC aging while little research has studied lysosomal dysregulation, proteasome declination and advanced glycation end products (AGEs).

### 3.4. Autophagy Defects and Autophagy Inducers

The autophagy system plays a crucial role in protein clearance. As a result of autophagy, dysfunctioned proteins and damaged organelles can be degraded into amino acids and reused by cells. Autophagy supports cell proteostasis under different situations, such as development and stress. Maintaining and inducing the normal function of autophagy has life-extending effects. Emerging evidence suggests that senescence and autophagy share overlapping signaling pathways. Typical autophagy inducers include inhibitors of TOR complex 1 (TORC1) (rapamycin), mitogen-activated protein kinase (MAPK) (spermidine) and PI3-kinase (quercetin), as well as activators of AMP-activated kinase (AMPK) (metformin) [81].

In MSC, autophagy plays an important role in MSC fate determination, bone remodeling and aging [82]. Autophagy induction can also be a self-defensive response against acute oxidative stress [83]. With age, Kynurenine (KYN) accumulates in BMSC and inhibits autophagy, promotes early senescence, and suppresses osteogenic differentiation [84]. A recent study demonstrated that autophagy flux was almost the same in both old and young BMSC, but there were remarkable ultrastructural differences [85]. An IGF-1 (insulin-like growth factor 1) knockdown decreased the Akt/mTOR signaling and increased autophagy in aged BM-MSCs exposed to hypoxia [86]. However, a previous study demonstrated that autophagy increases in BMSCs’ replicative senescence. A knockdown of p53 reduced autophagy and alleviated the senescence, accompanied by upregulated levels of mTOR and Rb phosphorylation [87]. This result suggests a difference between the mechanism regarding autophagy in the age-related and passage-related senescence of BMSCs. During human MSCs’ in vitro expansion, three-dimensional (3D) aggregation can reverse some of the adverse alterations of the 2D culture by heightening autophagy, leading to the rejuvenation of MSC [88]. Taken together, maintaining an optimal autophagy level may serve as an important strategy in preventing BMSC aging.

### 3.5. DNA Damage and Promotion of Genomic Stability

DNA damage of BMSC can be caused by a number of internal or external factors or diseases [80,89,90,91,92]. The accumulation of physical DNA damage and DNA damage response (DDR) activation have been reported in aged MSC at early passages [67]. It is reported that DNA damage drives accelerated bone aging via the NF-κB pathway [93]. The accumulation of DNA fragments and activation of DNA sensors and immune response could further trigger SASP secretion [94]. Several compounds have been reported to have protective effect against DNA damage in BMSC. Metformin inhibits DNA damage and senescence of BMSC induced by chronic kidney disease (CKD) and attenuates inflammation and fibrosis [95]. Zoledronate (ZOL) exerts a protective effect on MSC against DNA damage induced by ex vivo expansion and exposure to irradiation via inhibition of mTOR signaling [96]. Interestingly, low doses of isothiocyanates (ITCs) may contribute to attenuating the aging process related to oxidative DNA damage, while high concentrations may induce cytotoxicity and DNA damage [97].

### 3.6. Telomere Shortening

Early studies demonstrate that telomere length shortens during BMSC in vitro expansion and BMSC from old donors exhibited accelerated senescence and mean telomere length decrease [42,98,99]. The telomere length was significantly higher in BMSC from children compared with those from adults [100]. Telomerase deficiency led to an accelerated senescence of BMSC and impaired osteogenic differentiation capacity, leading to bone loss in mice [101]. When introducing the lentivirus-mediated dual expression of human telomerase reverse transcriptase (hTERT) and VEGF genes to modify human BMSCs from aged donors, prolonged life span and enhanced angiogenic ability of aged BMSCs were observed, suggesting a beneficial modification for BMSCs’ therapeutic effects [102]. Telomere shortness is involved in the senescence of BMSCs induced by H_2_O_2_ and prolonged passage. In the same study, olaparib was used to maintain the telomere length, resulting in attenuated SA-b-Gal staining and increased osteogenic differentiation [103].

### 3.7. Epigenetic Stress and Regulators

Cells undergo epigenetic changes such as DNA acetylation and methylation during aging. The modification of enzymes responsible for the epigenetic status significantly alters the aging process. Epigenetic clocks determine biological age based on individual DNA methylation levels. The reprogramming of youthful epigenetic information is proved to recover tissue function and promote regeneration in vivo [104]. Recent studies have also linked epigenetic modulations with aging in BMSC [105]. Sequencing-based methods reveal that the changes of DNA methylation dynamics related to aging is greater than previously reported [106]. The expression of epigenetic regulating enzymes usually decreases during aging. Senescence-escaped MSCs showed increased H3K9me and enhanced DNA methylation of the senescence-associated p16(INK4a) gene [107]. Recently discovered epigenetic regulators of both BMSC cell senescence and osteogenic differentiation includes nucleosome assembly protein 1-like 2 (NAP1L2), DNA N6-methyladenine (N6-mA) demethylase Alkbh1, KDM4B, H3K9 demethylases KDM3A and KDM4C, BMI1 [5,55,108,109,110,111,112]. Loss or gain of functions of these regulators affect BMSC self-renewal, senescence, and osteogenesis. RG108, a DNA methyltransferase inhibitor (DNMTi), is reported to have anti-senescence effects in human and porcine BMSC [113,114]. Besides direct modulation, melatonin stimulates the NSD2 (histone methyltransferase nuclear receptor binding SET domain protein 2) expression to rebalance H3K36me2 and H3K27me3 modifications, increasing chromatin accessibility of osteogenic genes [115]. However, there are limited studies regarding histone modifications in aged BMSC and a lack of knowledge as to which pharmacological or environmental factors can be applied to prevent epigenetic changes during aging.

Butyrylation is a type of protein modification where a butyryl group is added to a lysine residue. There is emerging evidence to suggest that butyrylation may play a role in regulating cellular senescence. For example, studies have shown that butyrylation levels are decreased in senescent cells, and that butyrylation of specific proteins can affect the expression of genes involved in senescence. Butyrylation has been shown to have important epigenetic effects, particularly with regard to the modification of histone proteins [116,117]. Bianchi et al. found that the butyrylation of high mobility group 1 (HMGB1) was decreased in senescent cells, and that butyrylation of HMGB1 reduced its ability to promote senescence [118]. Additionally, butyrate, a molecule that can promote butyrylation, has been shown to delay senescence in certain cell types [119]. Butyrate has also been shown to affect DNA methylation, another important epigenetic modification, and may play a role in regulating gene expression through this mechanism [120].

### 3.8. Noncoding RNAs (miRNA and LncRNA)

Bioinformatics and previous studies have shown that microRNAs (miRNAs) and other non-coding RNAs are important epigenetic regulators of senescence and aging in BMSC [121,122,123,124,125,126,127]. Recent studies have identified several non-coding RNAs during MSC aging with the aim of reversing this process. MiR-34a overexpression in young MSCs resulted in senescence features. Conversely, miR-34a suppression contributed to diminished senescence features in both replicative and natural senescent BMSCs by targeting Nampt and by the NAD+-Sirt1 pathway [128]. The results are in line with a previous study demonstrating that miR-34a plays pro-senescence roles in BMSCs by targeting Sirt1 [129]. Moreover, muscle-derived extracellular vesicles (EVs) containing elevated levels of the senescence-associated microRNA miR-34a decreased BMSC viability and increased BMSC senescence [130]. The miR-183 cluster (miR-96/-182/-183) is also found highly expressed in aged EVs, which inhibited the osteogenic differentiation of young BMSCs in vitro [131]. The level of miR-29b-1-5p was lower in young hBMSCs, but high in hBMSCs from the older patients. An overexpression of miR-29b-1-5p significantly reduced the osteogenic differentiation in younger BMSCs, and inhibitors to miR-29b-1-5p stimulated an osteogenic differentiation in older BMSCs [132]. Similar trends are also observed in miR-141-3p, miR-206 and miR-188 [133,134,135]. A recent study also reported that accumulation of kynurenine exerted age-related changes in BMSCs by altering microRNA profiles [136]. It has been reported that long noncoding RNA-p21 modulates BMSCs senescence via the Wnt/β-catenin signaling pathway [126].

### 3.9. Mitochondrial Dysfunction, Energy Metabolism and Mitochondrial Function Regulators

Accumulation of mitochondrial DNA (mtDNA) mutations and an elevated ROS level are major causes for mitochondrial dysfunction. Mitochondrial dysfunction impairs the nutrient sensing, energy homeostasis, and differential abilities of BMSCs [137]. Key regulators include the adenine monophosphate–activated protein kinase (AMPK) pathway, the phosphoinositide 3-kinase (PI3K)–AKT pathway, FoxO transcription factors, peroxisome proliferator-activated receptor gamma coactivator 1 α(PGC-1α) and sirtuin (SIRT) [138,139,140,141,142]. SIRT1 and SIRT6 expression decrease in aged BMSCs [143]. Sirt1 or Sirt6 deficiency increases BM-MSCs senescence and decreases bone mass [144,145]. PGC-1α and Sirt1 are involved in not only energy metabolism but also the regulation of BMSC differentiation into osteoblasts and adipocytes [146,147]. Phosphocreatine (PCr) promoted the osteoblastic differentiation, suppressed reactive oxygen species (ROS) over-generation and promoted the ATP production, which might be regulated by SIRT1/FOXO1/PGC-1α signaling pathway [138].

Mitophagy aims to eliminate the damaged and dysfunctional mitochondria. The accumulation of damaged mitochondria can lead to the deterioration of the stem cell function in the ageing process. Mitophagy plays a vital role in protecting BMSCs against oxidative stress [148]. MSCs are typically cultured in the atmospheric oxygen concentration (21% O_2_) which is at higher than physiological concentration (5% O_2_) [149]. Cellular responses are sensitive to culturing oxygen concentration. While at physioxia (3–5% O_2_), MSCs displayed increased reparative and proliferative functions [150,151] normal “hyperoxia” culturing conditions could have accelerated their premature senescence [149]. A hypoxic environment is conducive to glycolysis coupled with reduced OXPHOS and helps avoid oxidative damage, which may protect the BMSCs from aging [152].

Evidence has suggested the dysfunction of metabolic activity of BMSCs in aged mice. Aging BMSCs have depleted NAD(P)H, decreased oxidative phosphorylation and glycolytic activity, lower mitochondrial membrane potential, and decreased ATP production [137,153,154]. Several methods have been developed to combat NAD+ depletion. An overexpression of nicotinamide mononucleotide adenylyl transferase 3 (NMNAT3) increases the level of nicotinamide adenine dinucleotide (NAD+) and improves mitochondrial function in BMSCs [155]. Nicotinamide phosphoribosyltransferase is also reported to be able to postpone BMSCs’ senescence by mediating NAD(+)-Sirt1 signaling [156].

## 4. Biomarkers of Senescent BMSCs in Primary Cell Culture

Identifying senescent MSCs in primary culture is of significant scientific and therapeutic value, especially since cell-based regenerative therapy requires an ex vivo expansion of donor-derived MSCs. As mentioned above, proliferation arrest, DNA damage, telomere attrition, epigenetic changes, transcriptional and metabolic changes are all features that can be detected in vitro to identify senescent BM-MSCs. Current most frequently used methods include: the observation of enlarged and flattened cell morphology; cell cycle arrest; increased SA-β-gal activity; increased expression of p53, p21, and p16; shortened telomeres; SASP and DNA-scars and senescence-associated heterochromatin foci (SAHF) [157]. Here we present a summary including the most frequently used assays in BM-MSCs to assess senescence (Table 1).

## 5. Role of MSC Senescence in Age-Related Bone Diseases

Senescent cell accumulation and stem cell exhaustion with age impair the osteogenic supportive capacity and jeopardize the regenerative potential of BMSCs, impairing the recovery from skeletal injuries. SASP of senescent MSCs contribute towards accumulating cytokines to render the aged bone marrow increasingly pro-inflammatory [167]. The exacerbated inflammation in the marrow microenvironment, such as elevated transcription factor nuclear factor kappa B (NF-κB) signaling, would promote osteoclast differentiation, while potently inhibiting osteoblastic bone formation [32]. During aging, the loss of the self-renewal capacity and the osteogenic differential ability in BMSCs leads to impaired bone formation, as the balance of bone remodeling is further uncoupled in the inflammatory microenvironment [20,44]. Mounting in vivo evidence in rodents suggests that age-induced oxidative stress may contribute to osteoporotic bone loss [3]. Both estrogen-deficiency via ovariectomy (OVX) in mice and rats and aging-related bone loss exhibited increased oxidative stress markers. The buildup of oxidative stress leads to the activation of NF-κB in various aging tissues. Hence, with a contribution from senescent cells, age-exacerbated inflammation of the bone microenvironment could be a unitary driving force in the pathogenesis of osteoporosis.

Several lines of evidence suggest a dysregulated cell fate of MSCs could be another pathogenic mechanism for osteoporosis and skeletal aging [5,55,168]. The deterioration of the osteogenic activity of BMSCs with age is responsible for the loss of bone-forming efficiency in vivo. Meanwhile, increased adipose tissue accumulation in the bone marrow is attributed to aberrant lineage allocation of MSCs. Histone demethylase KDM4B favors osteogenesis over adipogenesis from human MSCs, by removing gene-silencing H3K9me3 chromatin marks on the promoters of osteogenic master regulator genes [55]. H3K9me3 expression is elevated in BMSCs of aged and OVX mice. Furthermore, the MSC-specific depletion of *Kdm4b* exacerbated skeletal aging and bone-fat imbalance in aged and OVX mice [5]. Intriguingly, KDM4B also plays a critical role in mediating and prevention of MSC senescence, while loss of KDM4B with age has been shown to accelerate loss of self-renewal and the premature onset of senescence [5,112]. These implicate the potential epigenetic link between MSC senescence and the bone-fat imbalance in skeletal aging.

The healing capacity of bone fractures is often compromised by age. Carvalho et al. demonstrated that BMSCs from older patients (60 and 80 years old) had impaired proliferative and osteogenic capacities compared to BMSCs from younger patients (30 and 45 years old) [71]. In mice, aged BMSCs lose osteochondrogenic activity and promote enhanced bone resorption by generating an inflammatory and pro-osteoclastic environment, which leads to a defect in the healing of bone fractures [33].

In the context of periodontitis, aging, hyperglycemia and bacterial lipopolysaccharide (LPS) induce cellular senescence in gingival fibroblasts and macrophages to aggravate the adaptive immune response and periodontal inflammation [2,7,22]. A persistent gram-negative bacterial infection could induce significant DNA damage and trigger premature senescence in alveolar bone cells, via p53 activation [169,170]. Further, SASP and oxidative stress compound the inflammatory microenvironment in the periodontium, leading to exacerbation of the host immune response that may attribute to the increased severity of periodontitis in elderly patients.

## 6. Therapeutic Approaches to Rejuvenate Aged MSCs

### 6.1. Genetic Reprograming

The rejuvenation of MSCs can be achieved by various genetic reprogramming techniques, including the transfection of synthetic self-replicating RNAs, the overexpression of telomerase and the re-differentiation of induced pluripotent stem cells (iPSCs) reprogramed from MSCs [Table 2]. The aging-related cellular phenotypes such as mitochondria function and membrane integrity could be reversed through de-differentiation into a pluripotent state, often through transcriptive reprogramming via overexpressing the key stemness markers including octamer-binding transcription factor 4 (Oct4), the sex-determining region Y-box 2 (Sox2) and kruppel-like factor 4 (Klf4) [171,172]. Noguchi et al. reported that the transfection of Venezuelan Equine Encephalitis (Vee)-reprogramming factor (Rf) RNA replicon (SR-RNA) co-expressing Oct4, Sox2 and Klf4 can be used to generate the reprogramed iPSCs with high proliferation ability [173]. However, while these iPSCs reprogrammed from MSCs resemble the profiles of somatic/embryonic stages, this re-establishment of the self-renewal and pluripotent stage is not a viable therapeutic strategy in vivo, as the direct injection of pluripotent cells leads to cancer and teratoma formation in mice [174,175]. A potential solution is the ex vivo generation of induced MSCs (iMSCs) from iPSCs which is obtained from reprogramed MSCs [176]. The general principles are as follows: (1) Generation of iPSCs from MSCs. This procedure requires the stable transfection of a series of epitomal plasmids carrying Oct4, Sox2, Klf4, c-Myc, Nanog and Lin28 [177]. (2) Generation of iMSCs via re-differentiation of iPSCs. The typical method is the culture of iPSCs in an MSC-specific medium containing 10% platelet lysate [178]. This method can be used to fully rejuvenate aged MSCs, not as partial rejuvenation in other methods, because all senescence and age-related DNA methylation are removed.

The bioactivity of telomerase is a key factor indicating the senescence and regenerative properties of the MSCs. An alternative reprogramming strategy is to overexpress telomerase reverse transcriptase (TERT), or specifically high levels of the catalytic subunit of telomerase, by lentiviral vectors encoding TERT. The genetically modified MSCs showed enhanced proliferation and delayed apoptosis, demonstrating the successful rejuvenate of aged MSCs [179].

### 6.2. Small Metabolites

Small metabolites are good candidates to rejuvenate aged MSCs to avoid the risk of carcinogenesis or off-target side effects caused by viral vectors or plasmids in genetic reprograming. The most direct idea is to use the small metabolite from cell lysates. EI-Badri et al. developed a method to prepare cell lysates from metaphase II (M II) oocyte [180]. The oocyte extract was obtained by simple ultrasonic treatment. The incubation of MSCs with different concentrations of oocyte extract can significantly rejuvenate the status of MSCs, especially the functions of mitochondrial, including mitochondrial localization, morphological changes, bioenergetics, transmembrane potential, and levels of the ROS. Alternatively, the rejuvenation of aged MSCs can be accompanied by the addition of either external or internal metabolites. Resveratrol (RSV) is a natural phytoalexin, which has been confirmed to exhibit rejuvenation effects in stem cells [181]. Jin et al. demonstrated that the delivery of RSV to MSCs in an inflammatory microenvironment can partially rescue the abilities of aggregate formation and osteogenic differentiation of MSCs [182]. They also found the effects of RSV rely on the Sirt1 and AMPK signaling pathway, in which the metabolic regulator PGC1α is the key factor. Nicotinamide adenine dinucleotide (NAD^+^) is essential for mitochondria function and cell metabolism. It has been reported that the addition of NAD^+^ precursor nicotinamide riboside (NR) can significantly increase the intracellular level of NAD^+^ [183]. Lian et al. demonstrated that the supplementation of NR can rescue MSCs from the senescence [73]. Furthermore, exogenous NAD(+) replenishment leads to increased intracellular NAD(+) levels and significantly postpones BMSC senescence by increasing the Sirt1 expression [184]. Nicotinamide mononucleotide (NMN), a key natural NAD(+) intermediate, effectively promoted osteogenesis and reduced adipogenesis of MSC, protecting bone from aging [185].

### 6.3. Antioxidants

Several antioxidants have been investigated in the anti-aging treatment for BMSC targeting ROS. Desferal^®^, an iron-chelating agent, was proved to be able to reduce ROS level, rejuvenate BMSCs from aged rats and alleviate age-related bone loss [186]. The epigenetic modulation of KAT6A through the Nrf2/ARE signaling pathway can also reduce ROS accumulation in aged BMSCs, thus promoting stemness of aging BMSCs [187]. Endogenous antioxidants include superoxide dismutase (SOD), catalase, glutathione, glutathione peroxidase, lipoic acid, bilirubin and ferritin. Sirt3 replenishment is reported to attenuate oxidative stress damage and rescue BMSC senescence by enhancing superoxide dismutase 2 (SOD2), leading to reduction of the cellular ROS level [188]. Coenzyme Q10, an endogenous lipophilic quinone ubiquitous, was able to inhibit BMSCs’ aging induced by D-galactose, via activation of MTOR signaling [189]. However, the role of glutathione and N-acetylcysteine (NAC), a ROS scavenger, have not been fully explored in BMSC aging. In BMSC expansion, a polydopamine (PDA)-coated substrate was used to scavenge extracellular ROS to prevent replicative senescence [190]. In addition, a recent study showed that culturing at low-density (LD, 50 cells/cm^2^) contributed to decreased level of the ROS and delayed MSC senescence [191].

However, most of these experiments are carried out in vitro. According to current views, an excessive exogenous supplementation of direct antioxidants can suppress the response of endogenous antioxidants and have pro-oxidative results [192,193]. Additionally, the efficiency of exogenous antioxidants to directly scavenge the ROS in vivo could be much lower and cannot be guaranteed compared to in vitro studies [192]. Thus, systemic supplementation of exogenous antioxidants may not easily achieve therapeutic effects in reversing MSC aging. A more beneficial way would be activating innate pathways that promote endogenous antioxidants production in vivo, such as the nuclear factor erythroid 2-related factor 2 (NRF2) pathway [194,195]. Chrysin is reported to promote bone regeneration in type 1 diabetic rats and protect BMSCs from high glucose-induced oxidative stress by PI3K/AKT/Nrf2 pathway [196]. Nevertheless, as older people have a higher risk of cancer development, another significant concern may be raised because of the potential effect of antioxidants on cancer promotion. A downside of NRF2 activation would be its protective effect on cancer cells against oxidative damage [197]. More in vivo evidence is needed in the application of antioxidants regarding BMSC aging.

### 6.4. Engineered Hydrogels

Manipulating the properties of natural or synthetic polymers, various properties of engineered hydrogels can be finely tuned, including biocompatibility, biodegradability, mechanical strength and responsiveness to stimuli such as temperature, pH or light [198]. Engineered hydrogels can either be employed to load anti-senescence cargos or used to mimic the functions of receptors in either cell-cell contacts or cell-ECM interactions. Li et al. designed a chitosan-based hydrogel to deliver the early-passage MSC-derived extracellular vesicles (EVs) to rejuvenate aged MSCs [199]. The MSC-derived EVs can significantly promote their proliferation and ECM production but inhibit the secretion of matrix metalloproteinases (MMPs), thereby serving as a potential therapeutic strategy to rejuvenate aged MSCs. N-cadherin is a transmembrane protein that plays an important role in cell adhesion and cell signaling, which mediates cell—cell adhesion by binding to other cadherin molecules on adjacent cells, thereby promoting the mechanosensing, proliferation and differentiation of MSCs [200]. Yang et al. developed a N-cadherin mimetic hydrogel rejuvenating and enhancing the chondrogenesis of MSCs via the regulation of the cell metabolism level, especially glycolysis and fatty acid oxidation [201]. Besides cell-cell contacts, cell-ECM interactions can also be recapitulated by engineered hydrogels. A growth factor-enriched microenvironment (GEM) is beneficial for the regenerative ability of MSCs. Liu et al. reported a sulfonated gelatin hydrogel which can significantly amplify bone morphogenesis protein-2 (BMP-2) receptor activation via enhancing the binding between BMP-2 and BMP-2 type II receptors (BMPR2) [202]. The amplified BMP-2 receptor activation enhanced the proliferation and osteogenesis of MSCs and partially rejuvenated the functions of aged MSCs.

### 6.5. Senolytics

The elimination of senescent cells (senolysis) is a promising strategy for anti-aging treatment. The genetic clearance of p16^Ink4a^ has been demonstrated to alleviate osteoporotic bone loss in aged mice [24]. The senolytic cocktail, dasatinib and quercetin (D+Q) treatment has been reported to be able to improve physical function and survival in mice [203]. Clinical trials for these senolytic strategies are underway, but it remains to be seen if the intermittent “hit-and-run” approach, whereby the least side effects could be achieved, would ensure a sustainable reduction of senescent cell burden [204]. It may also be likely that bioengineered delivery vehicles will need to be combined with the senolytic agents to optimize the dosage and efficacy of this strategy. Recently, Xing et al. reported quercetin delivered by hydrogel as an effective senolytic that locally eliminated senescent BM-MSCs in vitro and restored the self-renewal capacity as well as the osteogenic ability as a result [205].

**Table 2:** Summary of therapeutic approaches to rejuvenate aged MSCs.

Therapeutic Approaches	Strategies/Targets	Examples from Literature
Genetic reprogramming	Reverse telomere shortening	Overexpression of telomerase reverse transcriptase (TERT) [179]
	Re-differentiation of induced pluripotent stem cells (iPSCs) reprogramed from MSCs.	Generation of iMSCs via re-differentiation of iPSCs [176]
Small metabolites	Metabolites from young cell lysates	Metaphase II (M II) oocyte [180]
	Metabolites restoring mitochondrial function and oxidative stress	Resveratrol (RSV) [182], Nicotinamide riboside (NR) [73,184]
Antioxidants	Activation of signaling pathways that promote antioxidant production	CoQ10–MTOR [189], Chrysin–NRF2 [196]
Engineered hydrogels	Cell—cell contacts	N-cadherin mimetic hydrogel [201]
	Cell—ECM interactions	Growth factor-enriched microenvironment (GEM) mimetic hydrogel [202]
Senolytics	Elimination of senescent cells	Dasatinib and quercetin [203,204]

## 7. Concluding Remarks

Recent evidence suggests a strong link between MSC senescence and the progression of several age-related bone disorders. Beyond the intrinsic loss of proliferative and alteration in differentiation potentials, aging MSCs and SASP exacerbates the proinflammatory state of the tissue microenvironment. MSC senescence arises from multiple mechanisms such as the accumulation of oxidative stress, DNA damage and mitochondrial dysfunction. Targeting senescence and rejuvenation of aging MSCs are promising therapeutic paradigms that could alleviate several age-related bone diseases.

## Figures and Tables

**Table 1:** Markers of BM-MSC senescence and techniques for detection.

Methods	Detection Techniques	Senescent Features
Cell morphology	Microscopy	Enlarged and irregular cell shape [158]
CFU	Colony formation assay	Decreased CFU-fs number, showing decreased colony-forming efficiency, indirect method of assessing BM-MSC senescence [45]
Sa-β-gal	Microscopy (colorimetric activity assay with X-gal), Flow cytometry (fluorimetric activity assay with C_12_FDG), IF	Senescent cells appear blue under microscope [159]
8-oxo-dG	IHC, IF, ELISA	Marker of oxidative DNA or RNA damage [160]
γH2AX	IF, Flow cytometry, WB	Indirect measure of DNA double strand breaks [161]
Telomere length	qPCR-based telomere length analysis, Universal STELA	Shortened telomere lengths (directly correlated to replicative senescence, also occur during oxidative damage) [162]
Senescent markers at mRNA level	Real-time RT-PCR, Microarray, Bulk or single-cell RNAseq	p53 and cyclin dependent kinase inhibitors (p16 and p21) [124,163]
Senescent markers at protein level	WB, IHC, IF, Flow cytometry, LC-MS/ MS	p53, p16, p21, etc. [53,124,164]
SASP secretome	Real-time RT-PCR, WB, ELISA	Including growth factors and cytokines [67,165]
DNA methylation and other epigenetic markers	miRNAs, CpG sites, NGS after bisulfite treatment	Analysis of methylated cytosines [166]

CFU-f: colony-forming units-fibroblast (CFU-f) assay; SA-b-gal: senescence-associated b-galactosidase; X-gal: galactosidase substrate; IHC: immunohistochemistry; IF: immunofluorescence; WB: western blotting; ELISA: enzyme-linked immunosorbent assay; 8-oxo-gG: 8-Dihydro-8-oxo-2′-deoxyguanosine; γH2AX: phosphorylated H2A histone family member X; FISH: fluorescence in situ hybridization; PCR: polymerase chain reaction; RT-PCR: reverse transcriptase PCR; NGS: next generation sequencing.
